# Synthetic human leukemia models: towards precision medicine

**DOI:** 10.18632/oncotarget.22259

**Published:** 2017-11-02

**Authors:** Sonia Cellot, Brian T. Wilhelm, Frédéric Barabé

**Affiliations:** Frédéric Barabé: Centre de recherche en infectiologie du CHUL, Centre de recherche du CHU de Québec, Quebec City, Quebec, Canada; CHU de Québec Hôpital Enfant-Jésus, Quebec City, Quebec, Canada; Department of Medicine, Université Laval, Quebec City, Quebec, Canada

**Keywords:** human acute leukemias, xenograft models, engineered leukemias, pediatric leukemias, oncogenic fusions

The advent of high throughput sequencing technologies has helped to reveal the tremendous genetic heterogeneity that underlies acute myeloid leukemia (AML), including pediatric AML. This level of complexity had already been suspected given the variability in patient outcomes associated with standard chemotherapy regimens, composed essentially of a cytarabine and anthracycline backbone, irrespective of disease subgroup. Currently in children, cure rates can vary dramatically from above 90% for acute promyelocytic leukemia (PML-RARA fusion) to below 30% for high risk subgroups, such as selected MLL- (Mixed Lineage Leukemia) rearranged AML (e.g. MLL-AF6, MLL-AF10 fusions). The dramatic improvements in treatment outcomes obtained with the introduction of retinoic acid (ATRA) and arsenic trioxide (ATO) for the treatment of acute promyelocytic leukemia highlights the importance of tailoring therapies according to specific genetic AML subgroups. In the emerging era following the large scale sequencing of tumor genomes, engineered human models of high fatality pediatric AMLs will be seminal to identify disease subtype specific functional dependencies and develop novel therapeutic strategies. It is these models that will be required to overcome the paucity of primary human samples, the genetic heterogeneity of the disease, and the clonal evolution of commercially available cell lines.

In pediatric AML, translocations that generate chimeric fusion genes are critical transforming events and present in a high proportion of cases, including the highest risk categories. We have exploited the oncogenic potential of MLL fusions, considered hallmarks of infant and therapy-related AML, to generate human models of MLL-AF9 driven AML [1], using overexpression of the fusion in cord blood stem/progenitor cells (HSPC). Despite the recognition of MLL-AF9 as a dominant oncogenic fusion for more than 3 decades [2], the recent modeling of MLL-AF9 AML in the physiologically relevant context of human cells unravelled unappreciated disease specific biomarkers and functional susceptibilities. In this case, the receptor tyrosine kinase RET was shown to be selectively upregulated in MLL-AF9 AML, and shRNA-mediated knockdown of RET hampered leukemia cell growth, both *in vitro* and *in vivo*. In addition, a panel of leukemia activated genes was identified [3], a signature that can be further validated for minimal residual disease tracking, as these transcripts are expressed at high levels in the leukemia (compared to the MLL-AF9 fusion transcript itself), and usually not detected in normal bone marrow cells.

Human leukemia models will unmistakably become an indispensable complement to the currently developed model organisms used to gain further insights into relevant disease biomarkers and drug targets. For example, differences across species have been reported, especially in the cytological subtype of leukemia that is induced by a given oncogene, depending on the cell type that is used. Overexpression of MLL-AF4 in HSPC, for instance, gives rise to AML in mouse, but to acute lymphoblastic leukemia (ALL) when using adoptive transfer of engineered human cells into immunocompromised recipient mouse strains [4]. Similarly, model organisms were highly informative in determining the oncogenic potential and putative targets of the cytogenetically cryptic NUP98-KDM5A fusion, a recurrent aberration found in ∼10% of pediatric megakaryocytic AML (AMKL), a rare form of leukemia associated with dismal outcomes. Overexpression of NUP98-KDM5A was sufficient to transform mouse cells [5], giving rise to a CD34^+^CD117^+^ AML, but not to the CD34^-^CD41^+^CD61^+^ leukemic blasts typically seen in pediatric AMKL, possibly indicating distinct cells of origin. In addition, the anti-tumor activity of novel pharmacological compounds will need to be assessed in models that more accurately reflect human disease, as well as potential cross toxicity on the normal hematopoietic system. Clearly, the recent development of HSPC self-renewal agonists has shown considerable variability in responses between model organisms. As reported, the aryl hydrocarbon receptor inhibitor StemRegenin 1 (or SR1) can expand human, monkey and dog cells, but had no impact on mouse HSPC [6]. Hence human models are needed to confirm findings derived from animal models and cell lines, and to develop preclinical analytical pipelines to identify and test promising drug targets with a safe toxicity profile.

While human cells initially proved difficult to transform, the optimisation of human HSPC culture conditions and viral transduction protocols were critical milestones that paved the way to AML modeling. It is now possible to maintain engineered human AML cell lines in culture long-term (>100 days, personal data), setting the stage for large-scale *in vitro* genetic or chemical based screens. More restricted screens involving the most promising lead compounds (as single agents or in combination) can then be carried using AML subtype specific xenografts, to streamline pre-clinical testing, and only bring the most promising treatment modalities to clinical trials. A current inherent limitation of xenograft models, of course, remains the study of immune responses and bone marrow niche interactions, but improvements in this direction are actively being pursued [7, 8].

Overall, engineered human AML models constitute yet another weapon in our quest to conquer leukemia, that will enable us to tackle the heterogeneity of the disease with the identification of subtype specific functional susceptibilities, as well as improved biomarkers to gauge treatment responses. These models will be particularly critical for the rare and highly lethal AML subgroups, as seen in children, for which cure rates are stagnating and remain unacceptably low.
